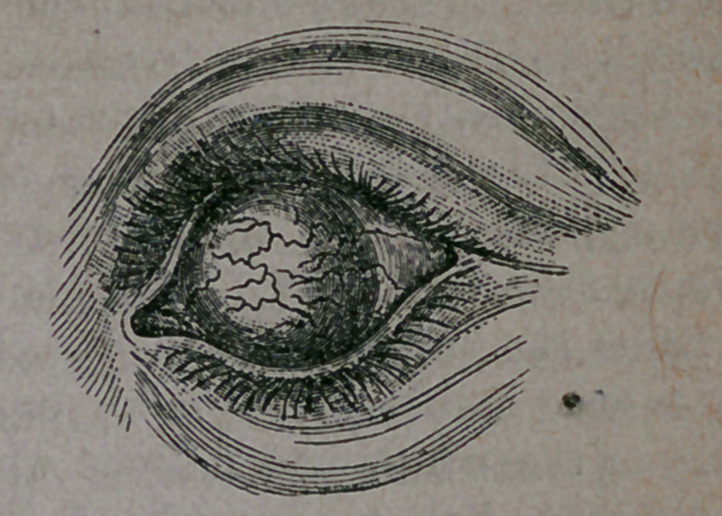# Staphyloma

**Published:** 1874-01

**Authors:** 


### STAPHYLOMA.

This deformity of the eye is frequently met with, and is one of the most unsightly to which the eye is subjected. It is a hernia or protrusion of the *cornea*, caused by neglected or uncontrollable inflammation of that structure, in which ulceration occurs, destroying the firmness of the cornea, and so allowing the humors behind it, to force it into a prominence far beyond the eye-lids. The cornea becomes pearly white, causing blindness; and from its exposed condition, protruding so far as to be unprotected by the eye-lids, is constantly subjected to attacks of inflammation, which in time, so annoys the well eye, as to greatly endanger it also. This appearance of the eye is well illustrated in the cut below, which will perhaps give a better idea of what is meant by *staphyloma*, than any description that can be written:

When the cornea protrudes as far as the yielding structure will allow, it ruptures, when an escape of the humors behind occurs, giving temporary relief of pain to the patient. The relief is of a short duration, however, for repeated attacks of pain and inflammation follow each other in such rapid succession as to compel the sufferer to seek relief, if possible, of some medical man.

Should the patient be fortunate enought to fall into the hands of a skillful oculist, he will at once recommend and execute an operation either of excision of the entire cornea, allowing a portion of the humors of the eye to escape, or still better, *extirpate* or remove the entire eye-ball, and so better adapt the socket to the insertion of an artificial eye.

This operation is speedily and easily executed, and is attended with no danger, affording prompt relief from pain, and restoring useful vision to the opposite eye; at the same time removing all cause of sympathetic inflammation which always occurs to the well eye in almost every case of disease of this nature,

## Figures and Tables

**Figure f1:**